# Single breath-hold 3D mapping of T1 and T2 relaxation times with 3D-QALAS - feasibility in patients

**DOI:** 10.1186/1532-429X-17-S1-W16

**Published:** 2015-02-03

**Authors:** Sofia Kvernby, Marcel Warntjes, Carl Johan Carlhall, Jan E Engvall, Tino Ebbers

**Affiliations:** 1 Institution for Medicine and Health Science, Linköping, Sweden; 2 CMIV, Linköping, Sweden

## Background

A novel method for 3D interleaved T1 and T2 mapping of the whole left ventricular myocardium within one breath hold, 3D-QALAS, has recently been developed [1]. The method has been evaluated both in phantoms and in healthy volunteers showing a good correlation to reference relaxation time mapping methods. The aim of this work was to investigate the feasibility of the method in patients with different cardiac pathologies and to extend the tested range of myocardial relaxation times by including measurements post injection of gadolinium (Gd) based contrast agents.

## Methods

In this feasibility study, eleven patients from an ongoing study were consecutively included (clinically significant aortic stenosis, ischemic cardiomyopathy and idiopathic dilated cardiomyopathy). The 3D-QALAS sequence which measures T1 and T2 relaxation times in 13 slices with a resolution of 2mm x 2mm x 6mm was run twice on a Philips Ingenia 3T scanner both pre and post Gd in all patients except for one patient were only native data was collected, due to poor renal function. For comparison, three (apical, mid-ventricular and basal) 2D 3-3-5 MOLLI acquisitions [2] were performed for myocardial T1 mapping pre- and post-contrast and three 2D multi-echo GraSE acquisitions were performed for T2 mapping pre-contrast. Ten healthy volunteers underwent the same acquisitions pre contrast as the patients.

## Results

**Figure 1:**
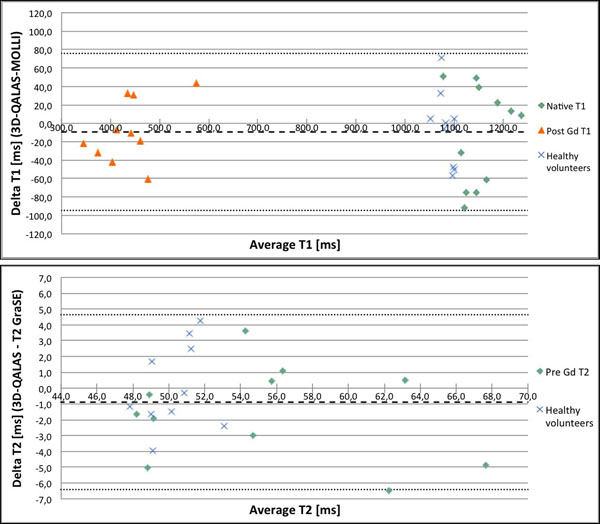
Upper: Bland-Altman plots for native and post contrast myocardial T1 relaxation times with 3D-QALAS and MOLLI. Dashed lines represent bias and ±2SD. Lower: Bland-Altman plots for native myocardial T2 relaxation times with 3D-QALAS and T2-GraSE. Dashed lines represent bias and ±2SD.

**Figure 2:**
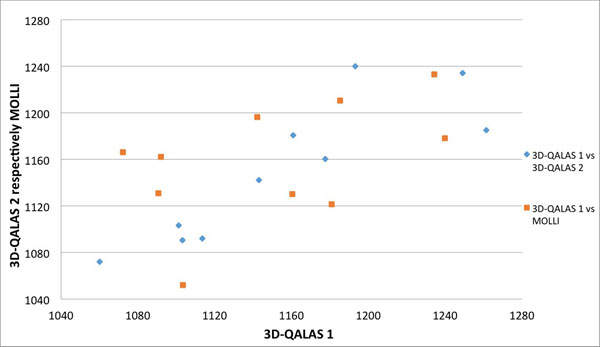
Correlation plot showing T1 values with 3D-QALAS measurement 1 versus 3D-QALAS measurement 2 and T1 values with 3D-QALAS measurement 1 versus MOLLI (3-3-5).

T1 and T2 relaxation times obtained with 3D-QALAS correlate well with reference methods, MOLLI for T1 (R=0,992) and Multi Echo GraSE for T2 (R=0,916). Data are visualized in Bland-Altman plots in figure 1. The overall average difference between measurements is -12,6 ms for T1 and -1,1 ms for T2. Myocardial mean T1 values pre-/post-contrast are 1140,8 ms /429,3 ms for 3D-QALAS and 1153,1 ms/442,2 ms for MOLLI. Myocardial mean T2 values pre-contrast are 54,1 ms with 3D-QALAS and 55,2 ms with Multi Echo GraSE. Correlation plot showing T1 values in the native range from 3D-QALAS measurement 1 vs. 3D-QALAS measurement 2 and 3D-QALAS measurement 1 vs. MOLLI can be seen in figure 2.

## Conclusions

The preliminary results from this ongoing study indicate that 3D T1 and T2 mapping in the left ventricle is feasible in one breath hold for patients with different pathologies using 3D QALAS. The method shows a good correlation in relaxation times measurement with existing 2D T1 and T2 mapping methods.

## Funding

Partly funded by the Swedish Heart and Lung Foundation.
